# Evaluation of Knowledge and Awareness of Dietary Nitrate Among Clinical Dietitians in Jeddah, Saudi Arabia: A Cross-Sectional Study

**DOI:** 10.7759/cureus.60597

**Published:** 2024-05-19

**Authors:** Reem Basaqr, Fatmah A Albathi, Abeer M Aljaadi, Abrar Babteen

**Affiliations:** 1 Clinical Nutrition, College of Applied Medical Sciences, King Saud Bin Abdulaziz University for Health Sciences, Jeddah, SAU; 2 King Abdullah International Medical Research Center, Ministry of the National Guard Health Affairs, Jeddah, SAU; 3 Clinical Nutrition, Faculty of Applied Medical Sciences, Umm Al-Qura University, Makkah, SAU

**Keywords:** saudi arabia, awareness, knowledge, assessment, dietary nitrate, dietitian

## Abstract

Background and objectives

Dietary nitrate (NO_3_) plays an important role in human physiological processes. In the past, inorganic NO_3_ was viewed negatively due to its link with carcinogenic effects, notably nitrosamine formation in the stomach; yet, current perspectives acknowledge NO_3_ as a potentially beneficial dietary element. Nutrition professionals (NPs) are crucial in promoting NO_3_ awareness in health and academic settings. The study aimed to evaluate the knowledge of NPs in Jeddah, Saudi Arabia, regarding the biological roles of dietary NO_3_, taking into consideration their qualifications and years of experience.

Methods

A cross-sectional study was conducted among NPs who had graduated from clinical nutrition programs or were employed in clinical or academic settings. A validated 12-item online questionnaire was used to assess dietary NO_3 _knowledge across five areas: health effects, dietary sources, recommendations, biomarkers of intake, and metabolism. The nitrate knowledge index (NKI) score was used to evaluate responses.

Results

Eighty-nine female NPs out of 144 completed the questionnaire. Most were ≤30 years old (75.4%) and had an undergraduate degree in clinical nutrition (70.8%), but 37 of them had ≤3 years of experience (62.7%). Overall, poor knowledge scores were observed among NPs, with a median (25^th ^and 75^th^ percentile) score of 10 (6, 13) out of 23. The majority (64%) perceived NO_3 _to be beneficial. However, most of the participants did not know its benefits in lowering blood pressure (BP) (68.5%) and were unsure about the effects of nitrate on cognitive function (60.7%) or kidney function (57.3%). Almost half of the NPs were unaware of NO_3 _sources and unsure about the mechanisms of the conversion of NO_3_ into nitrogen dioxide* *(NO_2_) in the mouth (48.3%). Overall, knowledge of factors that affect NO_3_ content in food was good. No significant differences were observed in the median NKI scores among the participants based on their level of education or years of experience.

Conclusion

This study suggests NPs lack knowledge about dietary NO_3_. To address this, educational programs should be developed and implemented in clinical and academic settings.

## Introduction

Dietary nitrate (NO_3_) is mainly obtained from plant-based sources [1,2]. Vegetables contain relatively high concentrations of NO_3_; thus, they can be considered a main source of NO_3 _intake in humans. Vegetables, specifically green leafy vegetables as well as beetroot, contribute approximately 85% of total NO_3_ intake [3]. The remainder comes from drinking water, or it is added as preservatives in the form of NO_3_ salts (i.e., sodium or potassium NO_3_) [3,4]. The amount of NO_3_ in different vegetables is extremely variable, and this is attributed to various environmental factors that may affect the concentration of NO_3_ content of vegetables from batch to batch, such as temperature, fertilizer use, and sunlight exposure [5].

In the past, inorganic NO_3_ had gained a negative reputation due to its association with perceived carcinogenic effects and the development of methemoglobinemia in infants, leading to its classification as a toxic component. Animal and epidemiological studies in the 1970s and 1980s have shown that high intake of dietary NO_3_ is associated with the formation of a carcinogenic compound called nitrosamine in the stomach [5] that acts as a precursor for the development of cancer. Therefore, the World Health Organization (WHO) Expert Committee on Food Additives and the European Food Safety Authority (EFSA) set the acceptable daily intake limit of dietary nitrate to 3.7 mg/kg; this is equivalent to 260 mg for a 70 kg adult [2,3]. However, this level can be easily exceeded in diets such as Dietary Approaches to Stop Hypertension (DASH) or the Japanese diet [3]. In addition, current reviews have confirmed a lack of association between dietary NO_3_ consumption and cancer risk [6,7]. Indeed, in 2010, the WHO Expert Committee concluded that there is insufficient evidence to establish any connection between an increase in NO_3_ consumption and the development of cancer [8]. Accordingly, there has been a shift in perspective regarding NO_3_ from being considered potentially harmful to being seen as a potentially beneficial dietary component.

Dietary nitrate is very well known as a substrate for the synthesis of the gasotransmitter nitric oxide (NO) [11], a master gaseous signaling molecule that has an array of functions in numerous human organ systems [9-14]. Therefore, many research studies have examined the therapeutic effects of dietary NO_3_ supplementation in a range of doses (~250-800 mg/day) among various populations [10,11,13]. Many studies have reported the beneficial effects of dietary NO_3_ supplementation, on oxidative stress, blood pressure (BP), endothelial function, exercise performance, and cognitive function [15]. Moreover, there is growing evidence that NO_3_-rich dietary patterns are associated with a lower risk of cardiovascular (CVD) and metabolic diseases [16,17]. In addition, there is an ergogenic effect of NO_3_ supplementation when consumed two to three hours before exercising, based on several studies [18-20].

Despite the known beneficial effects related to dietary NO_3_ intake, scarce data are available to demonstrate the level of knowledge of nutrition professionals (NPs) about dietary NO_3_ sources, effects, recommendations, and metabolism [21,22]. Recently, a group of researchers from the UK found that most NP participants have a good awareness of inorganic NO_3_ dietary sources and their physiological effects, particularly in improving sports performance and reducing BP. In addition, the authors demonstrated that the overall knowledge of participants with a graduate degree was more significant than that of those with an undergraduate degree [22].

It is crucial for NPs to maintain up-to-date knowledge of nutrition and dietary guidelines based on research. Such knowledge enables NPs to make informed healthcare decisions and greatly influences food choices and dietary behaviors. Additionally, the expertise of academics in dietary NO_3_ knowledge can positively be reflected in the students' learning outcomes. To our knowledge, no study has yet examined how much dietary NO_3_ information is disseminated to dietetic practitioners and academics in Jeddah, Saudi Arabia. Therefore, this cross-sectional study aims to evaluate the awareness of dietary NO_3_ and its beneficial effect among NPs in Jeddah, Saudi Arabia, based on their qualifications as well as their years of experience. This study may help the professions continue building a complex blend of knowledge and increase community awareness about the importance of a diet rich in dietary NO_3_ to improve patient health outcomes.

## Materials and methods

Participants

This study aimed to include a diverse group of NPs in Jeddah, Saudi Arabia. Clinical and sports dietitians with licenses from the Saudi Commission for Health Specialties working in public or private hospitals, sports facilities, or academic institutions were eligible to participate. The study also extended its recruitment to interns, postgraduate students, and qualified individuals with a clinical nutrition background who were currently unemployed. However, university academics working in food science departments and food scientists were excluded, in addition to all students who were enrolled in nutritional or dietetics courses at the undergraduate level. The participants were classified based on their qualifications as undergraduates or below (i.e., interns) and postgraduates, as well as their years of experience (>3 or ≤3 years). This study is designed to include no preference for gender, race, or ethnicity. Ethical approval was obtained from the Institutional Review Board of King Abdullah International Medical Research Centre, a research wing of King Saud bin Abdulaziz University for Health Sciences (KSAU-HS), Jeddah Campus, Jeddah, Saudi Arabia (approval number: SP21J/087/03).

Data collection

The Knowledge of Inorganic Nitrate Dietary Survey (KINDS), which has been developed and validated by Shannon et al., was used to collect data [22]. We started distributing our survey on September 12, 2021, until November 15, 2021. Prior to responding to the survey questions, participants were provided with a clear explanation of the survey's purpose. They were also requested to sign an online consent form as a means to ensure the validity and accuracy of their responses. To prevent duplicate submissions, the survey tool employs an automatic verification system. This system ensures that all questions must be fully answered before submission and prohibits participants from submitting the survey more than once. Potential participants in the targeted facilities were reached by distributing a questionnaire hyperlink via social media and email channels.

Survey tool and scoring method

The online study questionnaire (Appendix A) included a section dedicated to gathering demographic information. Participants were asked to provide details such as gender, age, hometown, employment status, name of hospital or workplace, type of hospital, years of experience, and educational level. Afterward, there were 12 questions classified into five parts, adapted from Shannon et al. [22]. Briefly, the questionnaire assessed participants' knowledge of dietary NO_3_, covering areas such as potential health and performance effects, intake values, dietary sources, biomarkers, and factors involved in the metabolism of inorganic NO_3_. A 23-point NO_3_ knowledge index was used to examine the participant’s responses to each question of the questionnaire. The scoring method used was similar to the original article, as follows: one point for the correct answer in each variable, and zero points for other answers, whether they are incorrect or unsure [22]. These scores were awarded for questions with clear evidence about the correct answers. However, the index does not include questions that reflect belief or opinion on inorganic NO_3_ where current evidence is ambiguous or there is no correct response (i.e., question three, all variables except sports performance and blood pressure, questions two, six, and 10).

Data management and analysis

Data are presented as n (%) or median (25^th^ and 75^th^ percentiles). Data were entered into Microsoft Excel (Microsoft Corp., Redmond, WA) and then exported and analyzed in Stata version SE/14.2 for Mac (Stata Corp., College Station, TX). Independent samples t-test or Wilcoxon rank-sum test were used to compare knowledge scores (continuous variables) between two groups, and chi-squared (χ2) test was used for comparison of proportions to compare differences in responses of subjects based on their education levels as well as their years of experience; Fisher's exact test was used when expected cell frequencies in the 2 × 2 table were ≤5. Clinical dietitians and academics were the two groups included in the years of experience analysis. The statistical significance was set at p <0.05.

## Results

Participant characteristics

**Figure 1:**
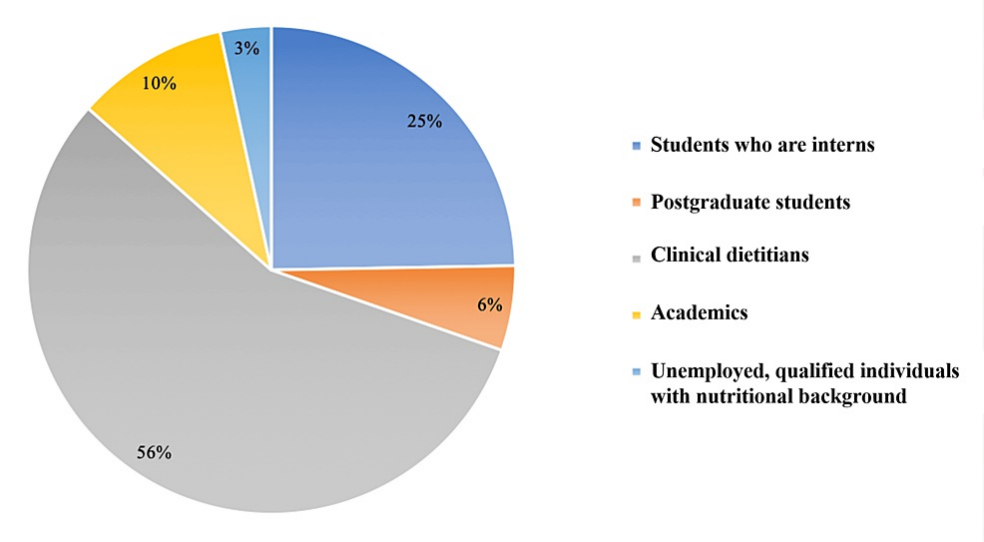
Distribution of jobs across nutrition professionals

Eighty-nine out of 144 (68.1%) female participants successfully completed the questionnaire. Demographic data are summarized in Table 1. Overall, the majority of them were aged ≤30 years (75.4%), possessed undergraduate degrees (70.8%), and were working as clinical practitioners (54.4%). Of those who were working as clinical practitioners (n = 59), 62.7% had ≤3 years of experience. The distribution of jobs across NPs is shown in Figure 1.

**Table 1:** Participants characteristics (n=89, all female) Data are presented as n (%); *Academics and clinical dietitians were the only groups included in the years of experience analysis

Participants' characteristics	n (%)
Age (years)	
≤30	67 (75.3)
31–60	22 (24.7)
Qualification	
Undergraduate degree or below	63 (70.8)
Master’s degree	20 (22.5)
Doctor of Philosophy degree (PhD)	6 (6.7)
Years of work experience*	
≤3 years	37 (62.7)
>3 years	22 (37.3)

Overall nitrate knowledge index (NKI)

**Figure 2:**
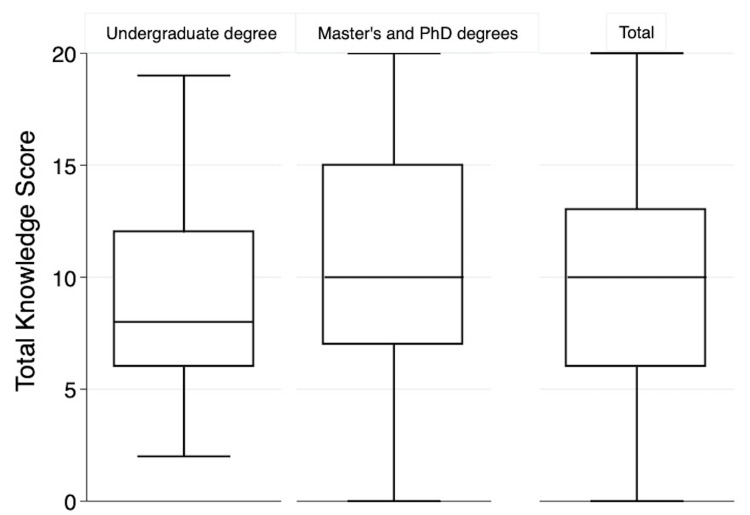
Overall score for nitrate knowledge for all nutrition professionals (NPs) and by qualification level Data are presented as median (25^th^ and 75^th^ percentiles). The total number of participants who responded to the questionnaire is n = 89 (undergraduate n = 63, higher qualification n = 26). A poor overall knowledge score was observed among NPs in most questions, as the median was 10 (6,13) out of 23. Values by qualification level were 10 (7,15) for higher qualifications and eight (6,12) for undergraduate degree holders.

Overall, poor knowledge was observed among NPs in most questions, with an overall median (25^th^ and 75^th^ percentiles) score for the NKI of 10 (6, 13) out of 23 (Figure 2). A substantial proportion of the participants (46.1%) had heard of dietary inorganic nitrate, and more than half believed that inorganic nitrate could be beneficial (64%). Although the majority of the participants held the belief that inorganic NO_3_ can have potential benefits, 68.5% of the participants were not aware that NO_3_ improves BP. Interestingly, 48.3% of the participants correctly identified the impact of dietary NO_3_ on sports performance. The participants demonstrated a generally good understanding of dietary sources of inorganic NO_3_ and the factors that influence the NO_3_ content of food. Approximately half of the NPs had correctly recognized some of the high sources of dietary NO_3_, such as spinach (48.3%) and beetroot (49.4%), whereas only 24.7% of the participants knew that radish is also a high source of NO_3_. Despite sausage and bacon being relatively low in NO_3_ content, a considerable number of participants (53.9% and 50.6%, respectively) identified them as a significant source of NO_3_. In addition, a noteworthy observation is that the majority of the participants (67.4%) provided the correct response regarding the content of NO_3_ in water, which is restricted to less than 50 mg/L. Generally, the participants exhibited limited knowledge regarding the acceptable daily intake (ADI) for this NO_3_. Specifically, 67.4% were unsure about ADI of NO_3_. A considerable number of participants displayed awareness regarding the influence of cooking (58.4%), pickling (47.2%), soil conditions (58.4%), and the use of fertilizers (53.9%) on the NO_3_ content of food, whereas a smaller proportion recognized the influence of season (30.3%) and storage conditions (36%). There was no consensus on the preferred biomarker for monitoring NO_3_ intake, with 36% choosing urinary NO_3_, 5.6% choosing salivary NO_3_, 6% selecting plasma, and 9% remaining uncertain. Regarding NO_3_ metabolism, 52.8% of participants could identify at least one correct compound (i.e., NO or nitrosamine). Furthermore, only 13.5% of the participants were aware that bacterial reductase is involved in the conversion of NO_3_ to nitrogen dioxide (NO_2_) in the mouth.

Knowledge difference based on qualifications

There was no significant difference in knowledge between NPs based on their levels of education (p >0.05), as shown in Table 2. A higher proportion of participants with master’s or PhD degrees (61.5%) were familiar with inorganic NO_3_, compared to participants with undergraduate degrees or lower (interns) (39.7%). Likewise, participants with postgraduate degrees demonstrated a higher likelihood of accurately identifying the physiological functions of NO_3_. For example, 53.8% of postgraduate degree holders recognized its positive impact on exercise performance, while 46.2% acknowledged its potential to reduce BP. Additionally, both groups, undergraduate and postgraduate degree holders, demonstrated limited understanding regarding the ADI for NO_3_, with approximately 67.6% and 68.2%, respectively, indicating uncertainty in this regard. Both undergraduate and postgraduate participants demonstrated similar levels of accuracy when identifying high sources of NO_3_. Approximately half of the participants in both groups successfully recognized certain foods, such as spinach (50.8% and 42.3%, respectively) and beetroot (49.2% and 50%, respectively), as being high in dietary NO_3_. However, a great number of both groups identified sausage (50.8% and 61.5%, respectively) and bacon (46% and 61.6%, respectively) as high sources of NO_3_. Regarding the understanding of the compounds resulting from the metabolism of NO_3_, participants holding an undergraduate degree or lower demonstrated a greater awareness (55.6%) of the compounds that inorganic NO_3_ converted into, including NO or nitrosamine, compared to postgraduate degree holders (46.2%). Participants with postgraduate degrees (30.8%) were found to have a higher likelihood of being aware of the exogenous pathway mechanism responsible for converting NO_3_ into NO_2_ in the mouth through oral bacterial reductase than participants with undergraduate degrees or lower (6.3%). A notable trend towards significance was observed in the knowledge gap regarding the impact of inorganic NO_3_ on glucose levels (p = 0.058), as most of the participants with undergraduate degrees appeared to be unsure of the dietary inorganic NO_3_ impact on glucose levels (61.5%). The median (25^th^ and 75^th^ percentiles) scores for the NKI for NPs who have undergraduate degrees were eight (6, 12) points out of 23, and for participants who have master's or PhD degrees, they were 10 (7, 15) points out of 23. The overall NKI did not significantly differ with levels of education (p = 0.2) (Figure 2).

**Table 2:** Nitrate knowledge in the overall cohort and when stratified by participant qualifications Data are presented as n (%). Postgraduates are those who have a master's degree or PhD. Significant p-values (<0.05) are shown in bold. *Questions that were included in the construction of the nitrate knowledge index (i.e., those where clear evidence exists for a correct answer). The correct answers are determined in italic type; each correct answer equals one point in the nitrate knowledge index. ‡ Participants who answered nitric oxide and nitrosamines got two points, and those who answered nitric oxide or nitrosamines got one point, who chose one of the other answers nitroglycerine, carbon dioxide, adrenaline, glucose, and unsure got 0 points

Question	Overall n (%)	Undergraduate or below n (%)		Postgraduate n (%)	P-value
Health and performance effects of dietary inorganic nitrate					
1. Have you heard of inorganic nitrate? *					0.150
Yes	41 (46.1)	25 (39.7)		16 (61.5)	-
No	23 (25.8)	19 (30.2)		4 (15.4)	-
Unsure	25 (28.1)	19 (30.2)		6 (23.1)	-
2. In your opinion is dietary inorganic nitrate a primarily beneficial or harmful nutritional component?					0.422
Beneficial	57 (64.0)	42 (66.7)		15 (57.7)	-
Harmful	32 (36.0)	21 (33.3)		11 (42.3)	-
3. For each of the following variables, please specify if it is increased or decreased by dietary inorganic nitrate:					
Sports performance*					0.682
Increased	43 (48.3)	29 (46.0)		14 (53.8)	-
Decreased	16 (18.0)	11 (17.5)		5 (19.2)	-
Unsure	30 (33.7)	23 (36.5)		7 (26.9)	-
Blood pressure*					0.159
Increased	35 (39.3)	27 (42.9)		8 (30.8)	-
Decreased	28 (31.5)	16 (25.4)		12 (46.2)	-
Unsure	26 (29.2)	20 (31.7)		6 (23.1)	-
Glucose levels					0.058
Increased	13 (14.6)	11 (17.5)		2 (7.7)	-
Decreased	15 (16.9)	7 (11.1)		8 (30.8)	-
Unsure	61 (68.5)	45 (71.4)		16 (61.5)	-
Lung function					0.503
Increased	24 (27.0)	18 (28.6)		6 (23.1)	-
Decreased	15 (16.9)	12 (19.0)		3 (11.5)	-
Unsure	50 (56.2)	33 (52.4)		17 (65.4)	-
Cancer risk					0.885
Increased	34 (38.2)	25 (39.7)		9 (34.6)	-
Decreased	12 (13.5)	8 (12.7)		4 (15.4)	-
Unsure	43 (48.3)	30 (47.6)		13 (50.0)	-
Cognitive function					0.908
Increased	19 (21.3)	13 (20.6)		6 (23.1)	-
Decreased	16 (18.0)	12 (19.0)		4 (15.4)	-
Unsure	54 (60.7)	38 (60.3)		16 (61.5)	-
Kidney function					0.825
Increased	17 (19.1)	13 (20.6)		4 (15.4)	-
Decreased	21 (23.6)	15 (23.8)		6 (23.1)	-
Unsure	51 (57.3)	35 (55.6)		16 (61.5)	-
Current and recommended intake values for nitrate					-
4. In the general population, what is the average daily intake of dietary inorganic nitrate of an individual? *					0.914
≤10 mg/day	15 (16.9)	10 (15.9)		5 (19.2)	-
11–50 mg/day	11 (12.4)	8 (12.7)		3 (11.5)	-
51–200 mg/day	7 (7.9)	5 (7.9)		2 (7.7)	-
201–500 mg/day	2 (2.2)	2 (3.2)		0 (0.0)	-
Unsure	54 (60.7)	38 (60.3)		16 (61.5)	-
5. Do you know what is the acceptable daily intake (ADI) of dietary inorganic nitrate? *					0.107
Currently no ADI	10 (11.2)	9 (14.3)		1 (3.8)	-
0.2 mg/kg body mass/day	5 (5.6)	2 (3.2)		3 (11.5)	-
3.7 mg/kg body mass/day	12 (13.5)	6 (9.5)		6 (23.1)	-
14.8 mg/kg body mass/ day	2 (2.2)	2 (3.2)		0 (0.0)	-
Unsure	60 (67.4)	44 (69.8)		16 (61.5)	-
6. In your opinion, does the ADI for dietary inorganic nitrate require revision?					0.941
Yes, it should be higher	6 (6.7)	4 (6.3)		2 (7.7)	-
Yes, it should be lower	12 (13.5)	9 (14.3)		3 (11.5)	-
No	5 (5.6)	4 (6.3)		1 (3.8)	-
Unsure	66 (74.2)	46 (73.0)		20 (76.9)	-
Dietary sources of inorganic nitrate					-
7. For the following foods, do you think they typically have a low (<50 mg/100 g fresh-weight) or high (>100 mg/100 g fresh-weight) dietary inorganic nitrate content?					
Spinach*					0.478
High	43 (48.3)	32 (50.8)		11 (42.3)	-
Low	23 (25.8)	14 (22.2)		9 (34.6)	-
Unsure	23 (25.8)	17 (27.0)		6 (23.1)	-
Sausage*					0.629
High	48 (53.9)	32 (50.8)		16 (61.5)	-
Low	23 (25.8)	17 (27.0)		6 (23.1)	-
Unsure	18 (20.2)	14 (22.2)		4 (15.4)	-
Tomato*					0.678
High	19 (21.3)	12 (19.0)		7 (26.9)	-
Low	39 (43.8)	29 (46.0)		10 (38.5)	-
Unsure	31 (34.8)	22 (34.9)		9 (34.6)	-
Beetroot*					0.987
High	44 (49.4)	31 (49.2)		13 (50.0)	-
Low	20 (22.5)	14 (22.2)		6 (23.1)	-
Unsure	18 (28.1)	18 (28.6)		7 (26.9)	-
Chocolate*					0.902
High	27 (30.3)	20 (31.7)		7 (26.9)	-
Low	29 (32.6)	20 (31.7)		9 (34.6)	-
Unsure	33 (37.1)	23 (36.5)		10 (38.5)	-
Bacon*					0.372
High	45 (50.6)	29 (46.0)		16 (61.5)	-
Low	19 (21.3)	14 (22.2)		5 (19.2)	-
Unsure	25 (28.1)	20 (31.7)		5 (19.2)	-
Lettuce*					0.193
High	24 (27.0)	19 (30.2)	5 (19.2)		-
Low	35 (39.3)	21 (33.3)	14 (53.8)		-
Unsure	30 (33.7)	23 (36.5)	7 (26.9)		-
Radish*					0.306
High	22 (24.7)	13 (20.6)	9 (34.6)		-
Low	23 (25.8)	16 (25.4)	7 (26.9)		-
Unsure	44 (49.4)	34 (54.0)	10 (38.5)		-
8. Which of the following factors do you think modify the inorganic nitrate content of food?					
Cooking*					0.905
Yes	52 (58.4)	37 (58.7)	15 (57.7)		-
No	12 (13.5)	9 (14.3)	3 (11.5)		-
Unsure	25 (28.1)	17 (27.0)	8 (30.8)		-
Season*					0.854
Yes	27 (30.3)	19 (30.2)		8 (30.8)	-
No	31 (34.8)	21 (33.3)	10 (38.5)		-
Unsure	31 (34.8)	23 (36.5)	8 (30.8)		-
Soil conditions*					0.420
Yes	52 (58.4)	35 (55.6)	17 (65.4)		-
No	9 (10.1)	8 (12.7)	1 (3.8)		-
Unsure	28 (31.5)	20 (31.7)	8 (30.8)		-
Use of fertilizer*					0.635
Yes	48 (53.9)	32 (50.8)	16 (61.5)		-
No	5 (5.6)	4 (6.3)	1 (3.8)		-
Unsure	36 (40.4)	27 (42.9)	9 (34.6)		-
Storage conditions*					0.463
Yes	32 (36.0)	23 (36.5)	9 (34.6)		-
No	17 (19.1)	10 (15.9)	7 (26.9)		-
Unsure	40 (44.9)	30 (47.6)	10 (38.5)		-
Pickling*					0.397
Yes	42 (47.2)	28 (44.4)	14 (53.8)		-
No	7 (7.9)	4 (6.3)	3 (11.5)		-
Unsure	40 (44.9)	31 (49.2)	9 (34.6)		-
9. How much dietary inorganic nitrate is there, on average, in drinking water? *					0.582
<50 mg/L	60 (67.4)	40 (63.5)	20 (76.9)		-
51–100 mg/L	18 (20.2)	15 (23.8)	3 (11.5)		-
101–200 mg/L	8 (9.0)	6 (9.5)	2 (7.7)		-
201–300 mg/L	3 (3.4)	2 (3.2)	1 (3.8)		-
Methods of evaluating inorganic nitrate intake					
10. Which biomarker would you choose to evaluate dietary inorganic nitrate intake?					0.981
Urinary nitrate	32 (36.0)	4 (36.5)	9 (34.6)		-
Salivary nitrite	5 (5.6)	3 (4.8)	2 (7.7)		-
Plasma nitrite	6 (6.7)	4 (6.3)	2 (7.7)		-
Exhaled nitric oxide	8 (9.0)	6 (9.5)	2 (7.7)		-
Unsure	38 (42.7)	27 (42.9)	11 (42.3)		-
Nitrate metabolism					
11. In the body, which of the following compounds is dietary inorganic nitrate converted into? *‡					0.679
0 point	37 (41.6)	25 (39.7)	12 (46.2)		-
1 point	47 (52.8)	35 (55.6)	12 (46.2)		-
2 points	5 (5.6)	3 (4.8)	2 (7.7)		-
12. Which one of these mechanisms is involved in the conversion of nitrate into nitrite in the mouth? *					0.035
C-reactive protein	4 (4.5)	3 (4.8)	1 (3.8)		-
Oxyhemoglobin	3 (3.4)	3 (4.8)	0 (0.0)		-
Salivary amylase	27 (30.3)	21 (33.3)	6 (23.1)		-
Bacterial reductases	12 (13.5)	4 (6.3)	8 (30.8)		-
Unsure	43 (48.3)	32 (50.8)	11 (42.3)		-

Knowledge difference based on years of experience

**Figure 3:**
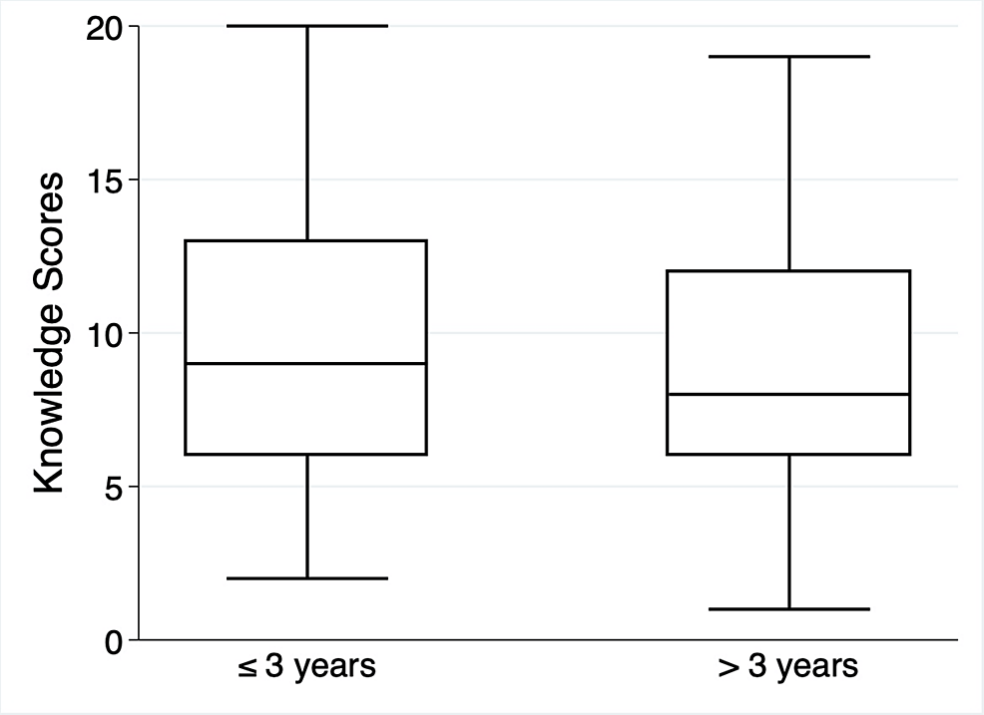
Overall scores for the NKI split by participants with ≤3 and >3 years of experience Data are presented as median (25^th^ and 75^th^ percentiles). The total number of participants who responded to the questionnaire is n = 89, ≤3 years n = 65, >3 years’ experience n = 24. The median for participants with ≤3 years was nine (6, 13), while participants with >3 years reported a median of eight (6, 12).

No significant difference in the knowledge of inorganic NO_3 _was observed based on years of experience across all questions (p >0.05), as shown in Table 3. A similar proportion of those with more than three years of experience (46%) and those with three years or less of experience (45.5%) have heard about inorganic NO_3_. Furthermore, the majority of both groups believed that inorganic NO_3_ was primarily beneficial (70.3% and 63.6%, respectively), and they were aware of its impact on improving sports performance (48.7% and 50%, respectively). While the results indicate a lower level of awareness among both groups of NPs (≤3 and >3 years of experience) (37.8% and 31.8%, respectively) regarding the role of NO_3_ in reducing BP, around half of the participants in both groups accurately identified certain foods, such as spinach (52.4% and 45.5%, respectively) and beetroot (48.7% and 45.5%, respectively), as being high in dietary NO_3_. However, a significant number of participants in both groups mistakenly identified sausage (40.5% and 68.2%, respectively) and bacon (43.2% and 54.6%, respectively) as high sources of NO_3_. Similarly, both groups of participants with (≤3 and >3 years of experience) showed comparable rates of correctly identifying at least one compound that NO_3_ can convert into, with proportions of 43.2% and 40.9%, respectively. Additionally, both groups demonstrated limited understanding regarding the ADI for NO_3_, with approximately 67.6% and 68.2%, respectively, being uncertain about it. The majority of participants in both groups (≤3 and >3 years of experience) answered the question about the amount of inorganic NO_3_ in drinking water correctly (67.7% and 66.7%, respectively). The results indicate poor overall knowledge regarding the mechanism of the conversion of NO_3_ into NO_2_ in the mouth by oral bacterial reductase, as only 12 participants correctly answered this question. About one-third of the participants with more than three years of experience (31.8%) knew the answer, compared to only 13.5% who had three years of experience or less of NPs. The median (25^th^ and 75^th^ percentiles) scores for the NKI for NPs who have three years of experience or less were nine (6,13) points out of 23, and for participants who have more than three years of experience, eight (6,12) points out of 23. The overall NKI did not significantly differ by years of experience (p = 0.9) (Figure 3).

**Table 3:** Nitrate knowledge in the overall cohort and when stratified by participant years of experience Data are presented as n (%). ≤3 years are participants who worked three years and less, >3 years are participants who worked more than three years. Significant p-values (p<0.05) are shown in bold. *Questions that were included in the construction of the nitrate knowledge index (i.e., those where clear evidence exists for a correct answer). The correct answers are determined in italic type; each correct answer equals one point in the nitrate knowledge index. ‡ Participants who answered nitric oxide and nitrosamines got two points, those who answered nitric oxide or nitrosamines got one point, and those who chose one of the other answers (nitroglycerine, carbon dioxide, adrenaline, glucose, or unsure) got 0 points.

Question	Overall n = 59 (%)	≤3 years n =37 (%)	>3 years n = 22 (%)	P-value
Health and performance effects of dietary inorganic nitrate				
1. Have you heard of inorganic nitrate? *				0.858
Yes	27 (45.8)	17 (46.0)	10 (45.5)	-
No	18 (30.5)	12 (32.4)	6 (27.3)	-
Unsure	14 (23.7)	8 (21.6)	6 (27.3)	-
2. In your opinion is dietary inorganic nitrate a primarily beneficial or harmful nutritional component?				0.598
Beneficial	40 (67.8)	26 (70.3)	14 (63.6)	-
Harmful	19 (32.2)	11 (29.7)	8 (36.4)	-
3. For each of the following variables, please specify if it is increased or decreased by dietary inorganic nitrate:				
Sports performance*				1.000
Increased	29 (49.2)	18 (48.7)	11 (50.0)	-
Decreased	10 (17.0)	6 (16.2)	4 (18.2)	-
Unsure	20 (33.9)	13 (35.1)	7 (31.8)	-
Blood pressure*				0.748
Increased	20 (33.9)	13 (35.1)	7 (31.8)	-
Decreased	21 (35.6)	14 (37.8)	7 (31.8)	-
Unsure	18 (30.5)	10 (27.0)	8 (36.4)	-
Glucose levels				0.840
Increased	8 (13.6)	6 (16.2)	2 (9.1)	-
Decreased	10 (17.0)	6 (16.2)	4 (18.2)	-
Unsure	41 (69.5)	25(67.6)	16 (72.7)	-
Lung function				0.721
Increased	17 (28.8)	11 (29.7)	6 (27.3)	-
Decreased	11 (18.6)	8 (21.6)	3 (13.6)	-
Unsure	31 (52.5)	18 (48.7)	13 (59.1)	-
Cancer risk				0.157
Increased	19 (32.2)	13 (35.1)	6 (27.3)	-
Decreased	7 (11.9)	2 (5.4)	5 (22.7)	-
Unsure	33 (55.9)	22 (59.5)	11 (50.0)	-
Cognitive function				0.751
Increased	14 (23.7)	10 (27.0)	4 (18.2)	-
Decreased	10 (17.0)	6 (16.2)	4 (18.2)	-
Unsure	35 (59.3)	21 (56.8)	14 (63.6)	-
Kidney function				1.000
Increased	12 (20.3)	8 (21.6)	4 (18.2)	-
Decreased	12 (20.3)	7 (18.9)	5 (22.7)	-
Unsure	35 (59.3)	22 (59.9)	13 (59.1)	-
Current and recommended intake values for nitrate				
4. In the general population, what is the average daily intake of dietary inorganic nitrate of an individual? *				0.416
≤10 mg/day	12 (20.3)	8 (21.6)	4 (18.2)	-
11–50 mg/day	5 (8.5)	2 (5.4)	3 (13.6)	-
51–200 mg/day	4 (6.8)	4 (10.8)	0 (0.0)	-
201 – 500 mg/day	1 (1.7)	1 (2.7)	0 (0.0)	-
Unsure	37 (62.7)	22 (59.5)	15 (66.7)	-
5. Do you know what is the acceptable daily intake (ADI) of dietary inorganic nitrate?*				0.212
Currently no ADI	4 (6.8)	4 (10.8)	0 (0.0)	-
0.2 mg/kg body mass/day	4 (6.8)	1 (2.7)	3 (13.6)	-
3.7 mg/kg body mass/day	11 (18.6)	7 (18.9)	4 (18.2)	-
14.8 mg/kg body mass/ day	0 (0.0)	0 (0.0)	0 (0.0)	-
Unsure	40 (67.8)	25 (67.6)	15 (68.2)	-
6. In your opinion, does the ADI for dietary inorganic nitrate require revision?				0.854
Yes, it should be higher	3 (5.1)	2 (5.4)	1 (4.6)	-
Yes, it should be lower	7 (11.9)	4 (10.8)	3 (13.6)	-
No	4 (6.8)	2 (5.4)	2 (9.1)	-
Unsure	45 (76.3)	29 (72.7)	16 (78.4)	-
Dietary sources of inorganic nitrate				
7. For the following foods, do you think they typically have a low (<50 mg/100 g fresh-weight) or high (>100 mg/100 g fresh-weight) dietary inorganic nitrate content?				
Spinach*				0.592
High	29 (49.2)	19 (52.4)	10 (45.5)	-
Low	12 (20.3)	6 (16.2)	6 (27.3)	-
Unsure	18 (30.5)	12 (32.4)	6 (27.3)	-
Sausage*				0.151
High	30 (50.9)	15 (40.5)	15 (68.2)	-
Low	15 (25.4)	11 (29.7)	4 (18.2)	-
Unsure	14 (23.7)	11 (29.7)	3 (13.6)	-
Tomato*				0.714
High	14 (23.7)	9 (24.3)	5 (22.7)	-
Low	22 (37.3)	15 (40.5)	7 (31.8)	-
Unsure	23 (39.0)	13 (35.1)	10 (45.5)	-
Beetroot*				0.467
High	28 (47.5)	18 (48.7)	10 (45.5)	-
Low	12 (20.3)	9 (24.3)	3 (13.6)	-
Unsure	19 (32.2)	10 (27.0)	9 (40.9)	-
Chocolate*				0.925
High	17 (28.8)	10 (27.0)	7 (31.8)	-
Low	17 (28.8)	11 (29.7)	6 (27.3)	-
Unsure	25 (42.4)	16 (43.2)	9 (40.9)	-
Bacon*				0.485
High	28 (47.5)	16 (43.2)	12 (54.6)	-
Low	12 (20.3)	7 (18.9)	5 (22.7)	-
Unsure	19 (32.2)	14 (37.8)	5 (22.7)	-
Lettuce*				0.556
High	16 (27.1)	12 (32.4)	4 (18.2)	-
Low	19 (32.2)	11 (29.7)	8 (36.4)	-
Unsure	24 (40.7)	14 (37.8)	10 (45.5)	-
Radish*				0.486
High	12 (20.3)	7 (18.9)	5 (22.7)	-
Low	12 (20.3)	6 (16.2)	6 (27.3)	-
Unsure	35 (59.3)	24 (64.9)	11 (50.0)	-
8. Which of the following factors do you think modify the inorganic nitrate content of food?				
Cooking*				1.000
Yes	30 (50.9)	19 (51.4)	11 (50.0)	-
No	10 (17.0)	6 (16.2)	4 (18.2)	-
Unsure	19 (32.2)	12 (32.4)	7 (31.8)	-
Season*				0.653
Yes	15 (25.4)	8 (21.6)	7 (31.8)	-
No	22 (37.3)	15 (40.5)	7 (31.8)	-
Unsure	22 (37.3)	14 (37.8)	8 (36.4)	-
Soil conditions*				1.000
Yes	33 (56.0)	20 (54.1)	13 (59.1)	-
No	4 (6.8)	3 (8.1)	1(4.6)	-
Unsure	22 (37.3)	14 (37.8)	8 (36.4)	-
Use of fertilizer*				1.000
Yes	34 (57.6)	21 (56.8)	13 (59.1)	-
No	1 (1.7)	1 (2.7)	0 (0.0)	-
Unsure	24 (40.7)	15 (40.5)	9 (40.9)	-
Storage conditions*				0. 588
Yes	21 (35.6)	15 (40.5)	6(27.3)	-
No	12 (20.3)	7 (18.9)	5 (22.7)	-
Unsure	26 (44.1)	15 (40.5)	11 (50.0)	-
Pickling*				0.518
Yes	29 (49.2)	18 (48.7)	11 (50.0)	-
No	5 (8.5)	2 (5.4)	3 (13.6)	-
Unsure	25 (42.4)	17 (46.0)	8 (36.4)	-
9. How much dietary inorganic nitrate is there, on average, in drinking water?*				0.921
<50 mg/L	38 (64.4)	23 (62.2)	15 (68.2)	-
51–100 mg/L	14 (23.7)	9 (24.3)	5 (22.7)	-
101–200 mg/L	5 (8.5)	4 (10.8)	1 (4.6)	-
201–300 mg/L	2 (3.4)	1 (2.7)	1 (4.6)	-
Methods of evaluating inorganic nitrate intake				
10. Which biomarker would you choose to evaluate dietary inorganic nitrate intake?				0.463
Urinary nitrate	18 (30.5)	10 (27.0)	8 (36.4)	-
Salivary nitrite	3 (5.1)	3 (8.1)	0 (0.0)	-
Plasma nitrite	3 (5.1)	1 (2.7)	2 (9.1)	-
Exhaled nitric oxide	4 (6.8)	2 (5.4)	2 (9.1)	-
Unsure	31 (52.5)	21(56.8)	10 (45.5)	-
Nitrate metabolism				
11. In the body, which of the following compounds is dietary inorganic nitrate converted into? *‡				0.841
0 point	30 (50.9)	19 (51.4)	11 (50.0)	-
1 point	25 (42.4)	16 (43.2)	9 (40.9)	-
2 points	4 (6.8)	2 (5.4)	2 (9.1)	-
12. Which one of these mechanisms is involved in the conversion of nitrate into nitrite in the mouth? *				0.444
C-reactive protein	1 (1.7)	1 (2.7)	0 (0.0)	-
Oxyhemoglobin	1 (1.7)	1 (2.7)	0 (0.0)	-
Bacterial reductases	12 (20.3)	5 (13.5)	7 (31.8	-
Salivary amylase	15 (25.4)	10 (27.0)	5 (22.7)	-
Unsure	30 (50.9)	20 (54.1)	10 (45.5)	-

## Discussion

This cross-sectional study aimed to assess the level of knowledge of dietary NO_3_ among clinical dietitians in Jeddah, Saudi Arabia. The key findings suggest that there was overall poor knowledge about NO_3_ among most participants, as the median NKI score was 10 out of 23 points. The majority perceived NO_3_ as beneficial, but many were unsure about its effects on BP and other physiological functions. More than half of our participants expressed uncertainty regarding the average population intake of NO_3_ and the ADI for this compound. In general, participants demonstrated a good awareness of some of the dietary sources of inorganic NO_3_ and some of the factors that can impact its presence in food. There was no significant difference in knowledge between NPs based on their levels of education or years of experience.

Despite the increase in the literature on the impact of dietary inorganic NO_3_ on various physiological functions, only half of the participants were aware of the positive impact of dietary NO_3_ on sports performance. However, the study demonstrated a general lack of knowledge regarding the impact of dietary NO_3_ on BP, despite the growing body of evidence on the beneficial effects of dietary NO_3_ in lowering BP and CVD health among healthy and clinical populations [12,23].

In contrast to our results, a study conducted in the UK has shown that NPs demonstrated greater awareness of the effect of inorganic NO_3_ on BP [22]. The discrepancy in awareness observed among participants, with a higher level of awareness regarding the positive impact of dietary NO_3_ on sports performance compared to BP regulation, may be attributed to individual differences in interests and exposure. Participants who possess a personal interest in sports or athletic performance may have proactively sought out information pertaining to nutrition and sports-related topics, leading to an elevated awareness, specifically in the context of sports performance and dietary NO_3_. In fact, enhancing the awareness of NPs as the primary educational resource for the public concerning the influence of NO_3_ on BP regulation brings forth numerous advantages. It empowers NPs to educate their clients more efficiently and incorporate the dietary NO_3_ sources in their nutrition medical plan, provide recommendations grounded in evidence for areas of practice in regards to NO_3_ benefits, remain up-to-date with the latest research, foster collaboration with other healthcare providers, help students identify the educational gap regarding this topic and broaden their knowledge in the future to understand the breadth of NO_3_ research, and actively engage in public health initiatives.

Most literature reports inconsistent evidence regarding the association between NO_3_ and the risk of cancer. In the 1970s and 1980s, animal and epidemiological studies indicated a correlation between high dietary nitrate intake and the formation of nitrosamine, a carcinogenic compound in the stomach. Nitrosamine acts as a precursor for cancer development [5]. However, it is important to note that most associations between nitrate and nitrite intake from red meat and the increased risk of gastric or colorectal cancer were relatively weak, typically less than two [4]. Recent research, such as the 2014 study by Dellavalle et al., suggests that colon cancer development may be more closely linked to reduced antioxidant levels, specifically vitamin C, than high nitrate levels [24]. The Shanghai Women's Health Study reported no association between NO_3_ intake and the risk of colorectal cancer [24]. Our study showed that most participants were either unsure of the physiological effect of NO_3_ on cancer risk or thought that dietary NO_3_ increased cancer risk. The WHO Expert Committee concluded that epidemiological and human evidence has failed to show any link between increased consumption of NO_3_ and cancer development [11]. Thus, increasing their knowledge about dietary inorganic NO_3_ sources would allow clinical practitioners to make recommendations to individuals without fear of cancer risk.

Our findings showed that most NPs were aware of NO_3_ from plant-based sources, but the majority believed that cured meats are also considered a primary source of NO_3_. While it was believed that cured meats, including bacon, ham, and hot dogs, are a high source of NO_3_, cured meat only contains less than 20 ng/100 g of fresh weight [25]. Participants may have simply assumed that NO_3_ sources rather than believing that certain vegetables are rich sources of NO_3_. Therefore, this misconception regarding the primary source of NO_3 _highlights the need for targeted education and accurate information dissemination among NPs, emphasizing the importance of differentiating between natural sources of NO_3_ found in green leafy vegetables and the potential health risks associated with excessive consumption of processed meats. Interestingly, most participants appeared to have good knowledge about the factors that may influence food’s NO_3_ content, such as soil conditions, cooking, and fertilizer. On the other hand, they were unaware that storage practices and seasoning methods impacted food’s nitrate content. Therefore, NPs' knowledge of environmental factors is essential to educating individuals on how to avoid the factors that might modify NO_3_ content.

Several researchers have raised concerns about the current ADI for NO_3_, which ranges from 0 to 3.7 mg/kg/day. Our findings revealed a general lack of knowledge in this area, as more than half of the participants were unsure about the ADI for dietary NO_3_ and if the ADI requires revision. As suggested by the previous paper conducted by Shannon et al. [22], raising awareness in this area is essential for NPs because it allows for more informed contributions to the ongoing debate on NO_3_ consumption and facilitates consensus on the need for potential revisions to the NO_3_ ADI. It also enables NPs to provide well-informed recommendations on NO_3_ intake, which is increasingly important given the growing interest in NO_3_ among researchers and the general public.

The mechanism of converting NO_3_ to NO_2_ occurs through oral bacteria called bacterial reductase via an exogenous pathway [5,26], and antibacterial mouthwash inhibiting this pathway has concomitant vascular effects in both the presence and absence of dietary NO_3_ intake [25,26]. However, most of our participants were unaware of this mechanism. Furthermore, approximately half of them were unsure that the inorganic NO_3_ compound converts to NO, nitrosamines, or both in the body. The NPs' knowledge in this area is essential to increasing their involvement in NO_3_ research. Without awareness of the exogenous (NO_3_- NO_2_-NO) pathway, NPs may struggle to grasp the underlying mechanisms and implications of studies investigating the effects of NO_3_ consumption. Thus, enhancing knowledge and awareness of this pathway is crucial for NPs to fully comprehend and apply NO_3_-related research in their professional practice.

Our findings revealed that, based on qualifications, there was no significant difference in knowledge among NPs, primarily due to the majority of participants holding undergraduate degrees. However, a study conducted by Shannon et al. in the UK demonstrated that participants with higher qualifications exhibited greater awareness across all aspects related to inorganic NO_3_ [22]. In contrast, our study conducted in Jeddah, Saudi Arabia, found a different trend. It indicated that participants with undergraduate qualifications displayed a higher likelihood of possessing better knowledge concerning inorganic NO_3_ compared to those with higher qualifications. These contrasting results highlight the influence of regional and cultural factors, emphasizing the importance of considering context-specific factors when examining the knowledge levels of NPs in relation to inorganic NO_3_.

Furthermore, our findings demonstrated that there was no significant difference in knowledge among NPs based on years of experience. However, there is a tendency for participants with less than three years of experience to show a greater level of inorganic NO_3_ knowledge than those with more than three years of experience. A possible reason is that the number of participants with more than three years of experience significantly varied compared to participants with less than three years of experience.

To our knowledge, this was the first study conducted in the Western region to evaluate knowledge and beliefs about dietary NO_3_ among NPs. Our study took a comprehensive approach by examining the potential differences in NO_3_ knowledge among NPs based on their qualifications and their years of experience. This approach allows for a more nuanced understanding of how knowledge may vary within the professional community. Despite the lack of significant differences, our findings indicate that both experienced and less-experienced NPs have limited knowledge of NO_3_. Therefore, there is a need for targeted educational interventions that focus on improving nitrate knowledge across the profession. These interventions can be designed to address common misconceptions, provide updated information, and enhance overall awareness. Increasing awareness about dietary NO_3_ is needed among NPs, especially those who work as practitioners, to make better-educated suggestions around NO_3_ intake for their clients who seek advice. Adequate nutrition knowledge about NO_3_ will highly improve the quality of life for patients with CVD and high BP, athletes, and the overall population. Therefore, NPs’ poor knowledge about NO_3_ intake will lead these patients to face more challenges.

There are a number of limitations to this study that must be taken into consideration. The small sample size may have limited our ability to observe significant differences in NPs' knowledge about NO_3_ based on qualifications and years of experience. Another limitation in data collection was that we could only reach a few relevant groups, despite the fact that the questionnaire was circulated through social media and universities. Therefore, our results may not be fully representative of the community of nutrition professionals.

Moreover, the questionnaire was too long, which led to losing some of our respondents in the middle of completing it. Also, our inclusion criteria were restricted to only NPs in clinical or academic fields, and we did not include any other fields related to nutrition, which might have contributed to these unexpected findings. While our current study is limited to the Western region of Jeddah city, expanding the research to different regions would provide a more comprehensive view and enable the generalization of findings throughout the country. This would enable a better assessment of the overall awareness levels and knowledge regarding NO_3_ across different populations in the country. For these reasons, our study may have been underpowered by the small sample size.

## Conclusions

In conclusion, this study reflected the poor knowledge and beliefs about inorganic NO_3_ among NPs in Jeddah, Saudi Arabia. Therefore, shedding light on the importance of dietary NO_3_ for subjects with cardiovascular risk factors and athletes may encourage NPs to continue developing research projects to tackle CVD with innovative dietary interventions. This illustrates the importance of disseminating knowledge to NPs through workshops in both clinical and academic settings, enabling them to better educate others about the benefits of dietary NO_3_.

## Appendices

Appendix A

Evaluation of Knowledge and Awareness of Dietary Nitrate Among Nutrition Professionals in Jeddah, Saudi Arabia: A Cross-Sectional Study

Demographic data

Gender

·      Female

·      Male

Hometown

·      Jeddah

·      Other

Qualifications

·      Bachelor

·      Master

·      PhD

·      Other

How long have you been working as a dietitian?

·      1-5 years

·      5-10 years

·      10-15years

·      Above 15 years

Name of the hospital

…………………………………………….

Do you work at a general/private hospital?

·      At the general hospital

·      At a private hospital

Employment status

·      DRT

·      RD

·      Senior RD

·      Nutrition support RD

·      Chief clinical RD

·      Department manager

Have you heard of inorganic nitrate?*

·      Yes

·      No

·      Unsure

Health and performance effects of dietary inorganic nitrate   

1. Have you heard of inorganic nitrate?    

·      Yes     

·      No      

·      Unsure                       

2. In your opinion is dietary inorganic nitrate a primarily beneficial or harmful nutritional component?

·      Beneficial                    

·      Harmful           

3. For each of the following variables, please specify if it is increased or decreased by dietary inorganic nitrate:

Sports performance  

·      Increased         

·      Decreased                    

·      Unsure                       

Blood pressure         

·      Increased                     

·      Decreased                    

·      Unsure                       

Glucose levels           

·      Increased         

·      Decreased                    

·      Unsure           

Lung function

·      Increased                     

·      Decreased        

·      Unsure           

Cancer risk    

·      Increased         

·      Decreased        

·      Unsure           

Cognitive function     

·      Increased         

·      Decreased        

·      Unsure           

Kidney function         

·      Increased         

·      Decreased        

·      Unsure           

Current and recommended intake values for nitrate    

4. In the general population, what is the average daily intake of dietary inorganic nitrate of an individual?   

·      10 mg/day      

·      11-50 mg/day            

·      51-200 mg/day          

·      201-500 mg/day        

·      Unsure

5. Do you know what is the acceptable daily intake (ADI) of dietary inorganic nitrate?         

·      Currently no ADI      

·      0.2 mg/kg body mass/day                 

·      3.7 mg/kg body mass/day                 

·      14.8 mg/kg body mass/ day

·      Unsure

6. In your opinion, does the ADI for dietary inorganic nitrate require revision?   

·      Yes-it should be higher      

·      Yes-it should be lower       

·      No      

·      Unsure                       

Dietary sources of inorganic nitrate         

7. For the following foods, do you think they typically have a low (<50 mg/100 g fresh-weight) or high (>100 mg/100 g fresh-weight) dietary inorganic nitrate content?

Spinach        

·      High   

·      Low    

·      Unsure           

Sausage       

·      High   

·      Low    

·      Unsure           

Tomato       

·      High   

·      Low    

·      Unsure           

Beetroot       

·      High   

·      Low    

·      Unsure           

Chocolate    

·      High   

·      Low

·      Unsure           

Bacon           

·      High   

·      Low

·      Unsure                       

Lettuce          

·      High   

·      Low    

·      Unsure                       

 Radish                     

·      High   

·      Low

·      Unsure                       

8. Which of the following factors do you think modify the inorganic nitrate content of food?

Cooking       

·      Yes     

·      No      

·      Unsure                       

Season         

·      Yes     

·      No      

·      Unsure           

Soil conditions 

·      Yes                 

·      No      

·      Unsure                       

 Use of fertilizer

·      Yes     

·      No      

·      Unsure           

Storage conditions      

·      Yes     

·      No      

·      Unsure           

Pickling                               

·      Yes     

·      No                  

·      Unsure                       

9. How much dietary inorganic nitrate is there, on average, in drinking water?

·      <50 mg/L                   

·      51-100 mg/L 

·      101-200 mg/L           

·      201-300 mg/L           

Methods of evaluating inorganic nitrate intake 

10. Which biomarker would you choose to evaluate dietary inorganic nitrate intake?

·      Urinary nitrate                       

·      Salivary nitrite           

·      Plasma nitrite 

·      Exhaled nitric oxide  

·      Unsure           

Nitrate metabolism  

11. In the body, which of the following compounds is dietary inorganic nitrate converted into?       

·      0 point            

·      1 point            

·      2 points          

12. Which one of these mechanisms is involved in the conversion of nitrate into nitrite in the mouth?        

·      C-reactive protein      

·      Oxyhemoglobin         

·      Salivary amylase      

·      Bacterial reductases   

·      Unsure
